# p73 coordinates with Δ133p53 to promote DNA double-strand break repair

**DOI:** 10.1038/s41418-018-0085-8

**Published:** 2018-03-06

**Authors:** Hongjian Gong, Yuxi Zhang, Kunpeng Jiang, Shengfan Ye, Shuming Chen, Qinghe Zhang, Jinrong Peng, Jun Chen

**Affiliations:** 1 Innovation Center for Signaling Network, College of Life Sciences, Zhejiang University, 866 Yu Hang Tang Road, Hangzhou, China 310058; 2 College of Animal Sciences, Zhejiang University, 866 Yu Hang Tang Road, Hangzhou, China 310058

## Abstract

Tumour repressor p53 isoform Δ133p53 is a target gene of p53 and an antagonist of p53-mediated apoptotic activity. We recently demonstrated that Δ133p53 promotes DNA double-strand break (DSB) repair by upregulating transcription of the repair genes *RAD51*, *LIG4* and *RAD52* in a p53-independent manner. However, Δ133p53 lacks the transactivation domain of full-length p53, and the mechanism by which it exerts transcriptional activity independently of full-length p53 remains unclear. In this report, we describe the accumulation of high levels of both Δ133p53 and p73 (a p53 family member) at 24 h post γ-irradiation (hpi). Δ133p53 can form a complex with p73 upon γ-irradiation. The co-expression of Δ133p53 and p73, but not either protein alone, can significantly promote DNA DSB repair mechanisms, including homologous recombination (HR), non-homologous end joining (NHEJ) and single-strand annealing (SSA). p73 and Δ133p53 act synergistically to promote the expression of *RAD51*, *LIG4* and *RAD52* by joining together to bind to region containing a Δ133p53-responsive element (RE) and a p73-RE in the promoters of all three repair genes. In addition to its accumulation at 24 hpi, p73 protein expression also peaks at 4 hpi. The depletion of p73 not only reduces early-stage apoptotic frequency (4–6 hpi), but also significantly increases later-stage DNA DSB accumulation (48 hpi), leading to cell cycle arrest in the G2 phase and, ultimately, cell senescence. In summary, the apoptotic regulator p73 also coordinates with Δ133p53 to promote DNA DSB repair, and the loss of function of p73 in DNA DSB repair may underlie spontaneous and carcinogen-induced tumorigenesis in p73 knockout mice.

## Introduction

The tumour repressor p53 plays a key role in the DNA damage response. Interestingly, p53 was shown to suppress DNA double-strand break (DSB) repair pathways, including homologous recombination (HR), non-homologous end joining (NHEJ) and single-strand annealing (SSA) [1–5]. ∆133p53 is an N-terminal truncated form of p53 with the deletion of both the MDM2-interacting motif and transactivation domain and part of the DNA-binding domain [6, 7]. *∆133p53* is directly transactivated by full-length p53 from an alternative *p53* promoter in intron 4 in response to both developmental and DNA damage stresses [8–11]. In turn, ∆133p53 antagonises p53-mediated apoptosis by differentially modulating the expression of p53 target genes [6, 12–14]. Zebrafish Δ113p53, a Δ133p53 orthologue, must interact with p53 to exert anti-apoptotic activity [15]. Basal Δ133p53 expression can inhibit p53-mediated replicative senescence in normal human fibroblasts, T-lymphocytes and astrocytes [16, 17]. Additionally, some cancer cells overexpress Δ133p53, which promotes angiogenesis and tumour progression [18]. However, ∆133p53 does not always inhibit the activity of full-length p53; under conditions of sub-toxic oxidative stress, ∆133p53 can coordinate with p53 to promote cell survival [19].

We recently revealed that upon γ-irradiation, Δ133p53 not only represses cell apoptosis, but also promotes DNA DSB repair by upregulating the transcription of DNA DSB repair-related genes such as *RAD51*, *RAD52* and *LIG4* [3]. Δ133p53 promotes the transcription of these three repair genes by binding to a novel p53 response element (RE) in the gene promoters independently of full-length p53. It remains unclear how Δ133p53 enhances the expression of the repair genes despite lacking the transactivation domain.

The p73 gene, a member of the p53 family, similarly encodes several isoforms. The N-terminal isoforms comprise two major groups, TAp73 (p73) and ΔNp73, which are transcribed from two promoters and have opposing cellular actions [20–24]. Full-length p73 and p53 share several target genes related to the control of cell cycle and apoptosis [25, 26]. However, p53 and p73 are not entirely functionally redundant, as both exhibit promoter selectivity and have a number of unique target genes. In mice, a loss of function of p73 leads to infertility and spontaneous and carcinogen-induced tumorigenesis, as well as hippocampal dysgenesis [27–29]. Although a previous chromatin immuno-precipitation (ChIP)-based analysis found that p73 binds to the promoters of some DNA DSB repair genes, such as *Rad51*, *Mre11* and *Brca2*, the overexpression of p73 did not greatly increase the expression of these genes [30]. These studies indicate that p73 plays a role in the maintenance of genomic stability. However, the mechanism by which p73 affects DNA damage repair remains incompletely understood.

Although full-length p53 does not interact with p73, Δ133p53 isoforms (including α, β, γ) were found to form complexes with p73 under conditions of overexpression. However, it remains unknown whether Δ133p53 affects the transcription activity of p73. Here, we demonstrate that upon γ-irradiation, Δ133p53 promotes DNA DSB repair by promoting p73 to bind to the promoters of repair-related genes such as *RAD51*, *RAD52* and *LIG4*. Additionally, we found a number of somatic mutations in the p53 REs of Δ133p53 promoter in different cancer tissues from the Catalogue of Somatic Mutations In Cancer (COSMIC) database. The mutations of C>A or >G at one of the mutated residues in exon-4 of full-length p53 do not change the codon for Thr amino acid in the full-length p53 protein, but the C is a very important consensus residue in the p53 RE. Through promoter analysis, we demonstrate that the mutations attenuated the activation of Δ133p53 upon DNA damage. Together, our data suggest that both of p73 and Δ133p53 are required to maintain genetic stability.

## Results

### Full-length p73 is activated in response to γ-irradiation

**Fig. 1:**
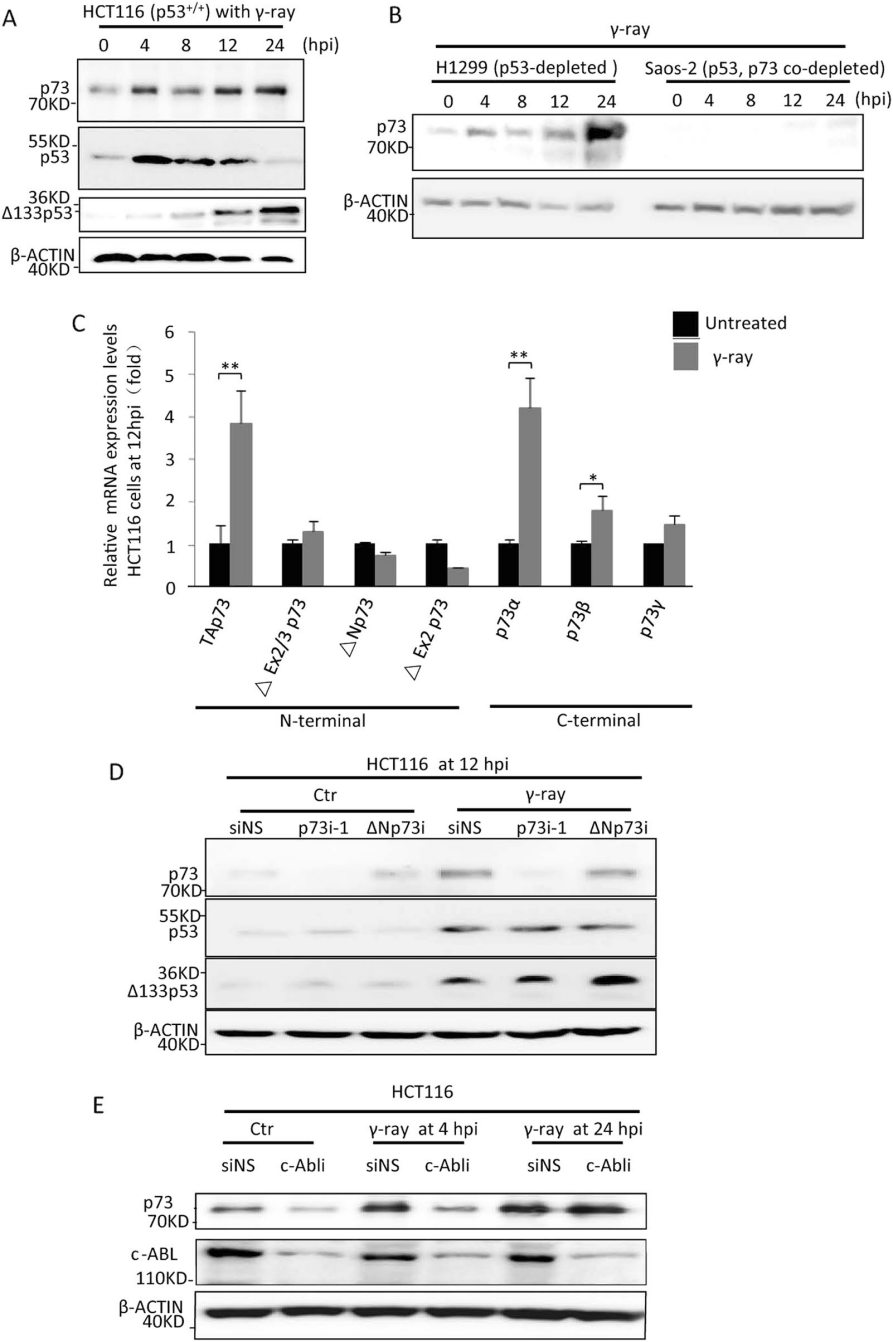
The activation of full-length p73 upon γ-irradiation. **a** Kinetics of p73, p53 and Δ133p53 protein expression in HCT116 (p53^+/+^ and p73^+/+^) cells treated with 10 Gy of γ-ray irradiation at 0, 4, 8, 12 and 24 h post irradiation (hpi). β-ACTIN was used as the protein loading control. **b** Kinetics of p73 protein in H1299 (p53^−/−^) and Saos-2 cells (lacking both endogenous p73 and p53 proteins) upon 10 Gy of γ-ray irradiation. **c** Relative transcript expression of p73 isoforms in HCT116 cells treated with 10 Gy of γ-irradiation as measured by quantitative real-time (qRT)-PCR at 12 hpi. Different isoform transcripts were amplified using a specific pair of primers as described in Fig. S1. Transcript expression was normalised against *β-ACTIN* and expressed as the fold change compared to the untreated control (Ctr). **d** Western blot analysis of p73, p53 and Δ133p53 expression in HCT116 cells transfected with non-specific siRNA (siNS), *p73*-interference RNA-1 (p73i-1) or *ΔNp73*-interference RNA (ΔNp73i), followed by 10 Gy of γ-irradiation at 12 hpi. **e** Western blot analysis of c-Abl and p73 expression at 4 and 24 hpi in HCT116 cells transfected with siNS or c-Abli, followed by 10 Gy of γ-irradiation

According to previous reports, p73 activation upon DNA damage occurs via two mechanisms. In the first, p73 is phosphorylated by c-Ab1 tyrosine kinase [31, 32]. In the second, p73 mRNA expression can be induced by the transcription factor E2F1, which is stabilised by Chk1 and Chk2 kinases [33–36]. To investigate the role of p73 in DNA DSB repair, we exposed HCT116 (p53^+/+^; p73^+/+^) human colorectal carcinoma cells to γ-irradiation. Western blots showed that the expression of both p53 and Δ133p53 proteins peaked at 4 h post irradiation (hpi) and 24 hpi, respectively, consistent with our previous results [3]. Interestingly, p73 protein exhibited a different accumulation pattern, with two peaks appearing at 4 and 24 hpi (Fig. 1a), which was also observed in p53-depleted H1299 human lung carcinoma cells, but not in Saos2 human osteogenic sarcoma cells lacking both endogenous p73 and p53 proteins (Fig. 1b).

Using various paired primers to amplify different N- and C-terminal isoforms of p73, we observed the most significant increase in the N-terminal full-length p73 transcript, rather than the ΔNp73 transcript, at 12 hpi. Among the C-terminal isoforms, the α-isoform transcript was increased by approximately fourfold, whereas the transcripts of β- and γ-isoforms were increased by less than twofold (Figure S1, Fig. 1c). Protein analysis also showed that p73 accumulation was depleted only by p73 siRNA (p73i-1), but not by ΔNp73 siRNA (ΔNp73i) (Fig. 1d). These findings demonstrate that γ-irradiation mainly activates full-length p73α. Additionally, p73 depletion did not influence Δ133p53 and p53 accumulation (Fig. 1d).

The quantitative real-time (qRT)-PCR analysis of cells at 3 hpi revealed that not all p73 isoforms were upregulated (Figure S2), suggesting that protein stabilisation led to the accumulation of p73 protein at 4 hpi. Protein analysis showed that c-Abl depletion obviously decreased the accumulation of p73 protein in irradiated cells at 0 and 4 hpi, but not at 24 hpi (Fig. 1e). Taken together, these results suggest that the two p73 protein peaks upon γ-irradiation could be attributed to different activation mechanisms.

Our observation that both p73 and Δ133p53 accumulate at 24 hpi led us to hypothesise that Δ133p53 may coordinate with p73 to promote DNA DSB repair.

### p73 and Δ133p53 act synergistically to promote DNA DSB repair

**Fig. 2:**
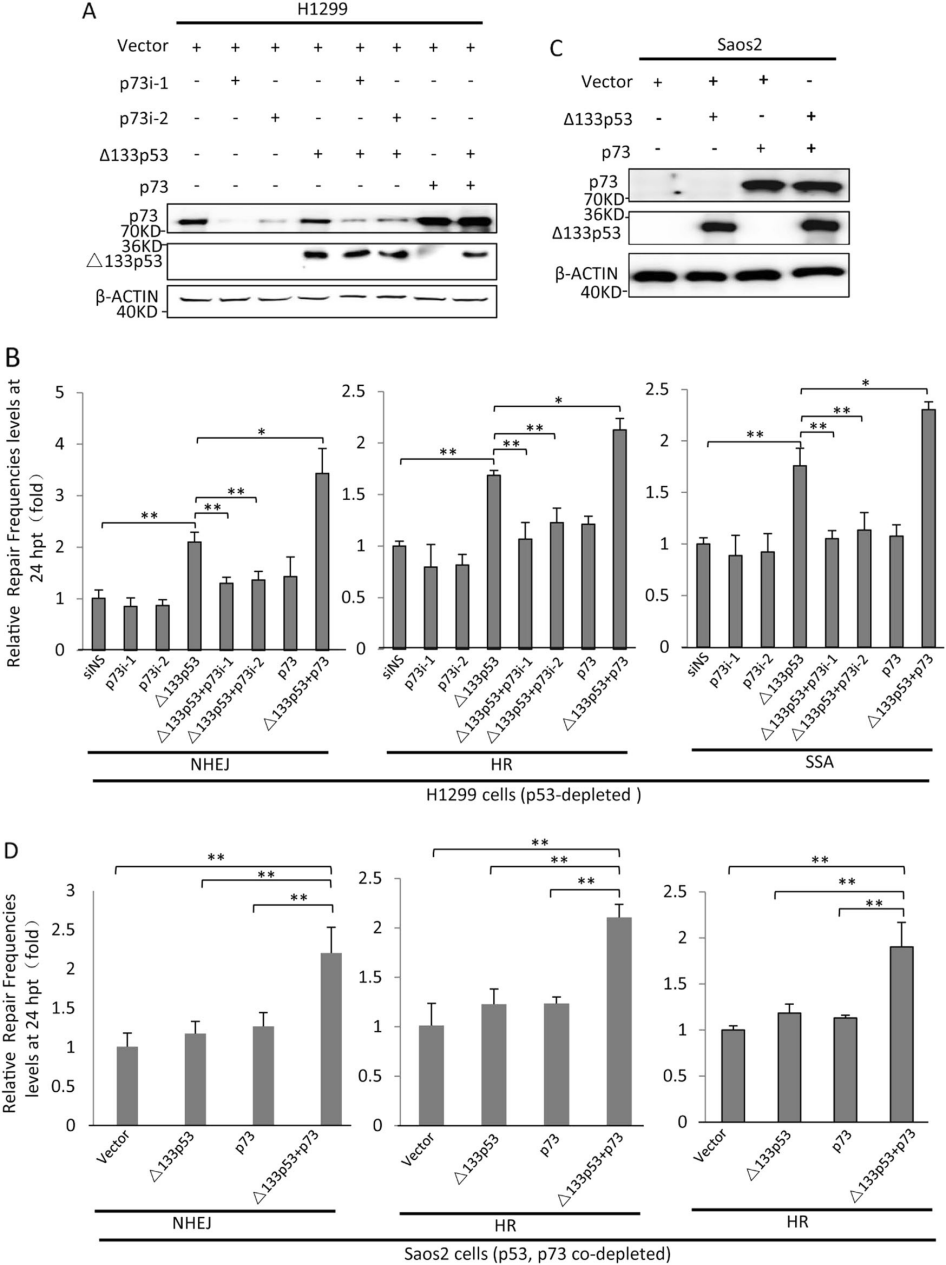
*p73* promotes homologous recombination (HR), non-homologous end joining (NHEJ) and single-strand annealing (SSA) repair pathways. **a**, **b** Effects of *p73* on HR, NHEJ and SSA repair frequencies in H1299 cells (p53^−/−^, p73^+/+^). The corresponding plasmids were linearised using I-*Sce*I. H1299 cells were transfected with constructs corresponding to each of the three repair assays and a non-specific siRNA control (siNS), two *p73* siRNAs [siRNA1 (p73i-1) or siRNA2 (p73i-2)], a *CMV-Δ133p53* plasmid, the *CMV-Δ133p53* plasmid together with each of the two p73 siRNAs or the *CMV-p73* or *CMV-Δ133p53* plasmid together with *CMV-p73* plasmids. Western blot of p73 and Δ133p53 expression in H1299 cells transfected with different reagents as indicated (**a**). The average repair frequencies were measured using a quantitative (q) PCR analysis of repaired assay constructs from three repeat experiments at 24 h post treatment (**b**). **c**, **d** Effects of *p73* on HR, NHEJ and SSA repair frequencies in Saos-2 cells (lacking both endogenous p73 and p53 proteins). Western blot of p73 and Δ133p53 expression in Saos-2 cells transfected with different reagents as indicated (**c**). The average repair frequencies were analysed as described for H1299 cells (**d**). All statistically significant differences between treatments were assessed using the independent-samples *T*-test (*P < 0.05, **P < 0.01)

To investigate whether Δ133p53 requires p73 to promote DNA DSB repair, we used H1299 cells and three EGFP-repairing-aided visual-plus-quantitative analysis reporter systems to measure HR, NHEJ and SSA repairs [37]. Consistent with our previous study, Δ133p53 overexpression led to significant increases in the efficiencies of the three DNA DBS repair pathways. Interestingly, this Δ133p53 overexpression-mediated increase in cellular DNA DSB repairs was impaired by the knockdown of p73, whereas either the knockdown or overexpression of p73 alone did not significantly affect the three DNA DSB repair pathways in these p53-deficient cells. However, the efficiencies of all three repair mechanisms increased further in cells co-transfected with p73 and Δ133p53, compared to those transfected with Δ133p53 alone (Fig. 2a, b).

The results were confirmed in Saos2 cells. The co-expression of Δ133p53 and p73 nearly doubled the efficiencies of all three DNA DSB repair pathways. However, the efficiencies of all three DNA DSB repair pathways were not significantly altered in cells expressing either Δ133p53 or p73 alone (Fig. 2c, d). Taken together, these data demonstrate that Δ133p53 and p73 depend on each other to promote DNA DSB repair.

### p73 promotes apoptosis during the early stage, but not the late stage, after γ-irradiation

According to previous studies, p73 plays a positive role in the induction of apoptosis upon γ-irradiation [26, 31, 35]. As p73 protein expression peaked twice at 4 and 24 hpi, we investigated whether both peaks contributed to the induction of apoptosis. The fluorescence-activated cell sorting (FACS) analysis of apoptosis with propidium iodide (PI) and Annexin V in HCT116 cells showed that p73 knockdown significantly decreased the proportion of apoptotic cells from 4 to 6 hpi, but not at 24 hpi (Fig. S3A, S3B). The data revealed that p73 promotes apoptosis at an early stage after γ-irradiation, but not at a late stage.

### p73 promotes the formation of DNA DSB repair foci and decreases DNA DSB accumulation upon γ-irradiation

**Fig. 3:**
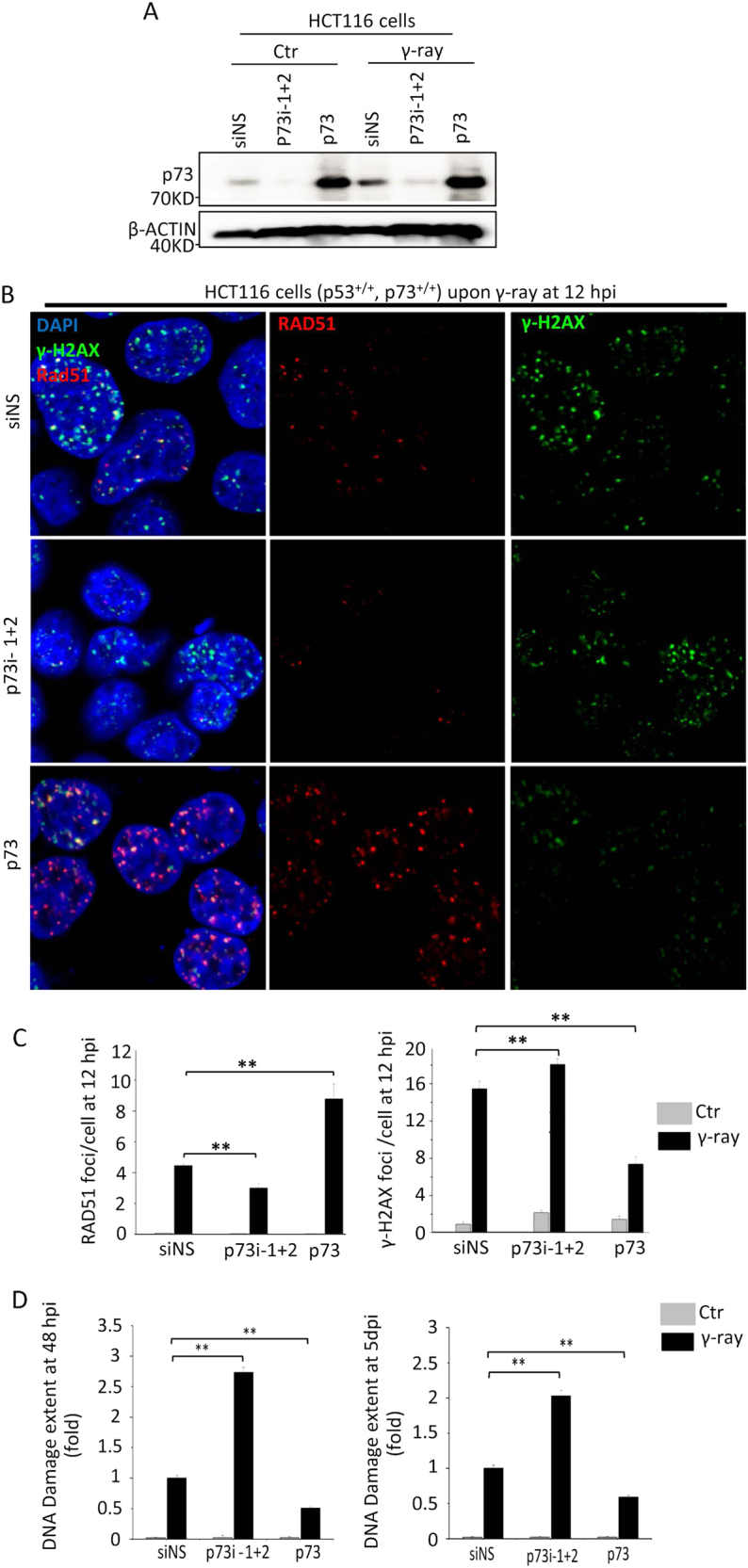
*p73* promotes the formation of RAD51 foci and DNA double-strand break (DSB) repair following ionising irradiation. **a** Western blot analysis of p73 expression in HCT116 cells transfected with non-specific siRNA (siNS), a mixture of *p73*-interference RNA-1 (p73i-1) and p73i-2 or *CMV-p73* plasmid, followed by 10 Gy of γ-ray irradiation. **b** Co-immunostaining of RAD51 (in red) and γH2AX (in green) in HCT116 cells subjected to different treatments as described in **a**. Specific monoclonal antibodies were used to evaluate RAD51 and γH2AX focus formation at 12 hpi as indicated. DAPI was used to stain nuclear DNA (blue). **c** Statistical analysis of the average numbers of RAD51 and γH2AX foci per cell in different samples, as shown in **b**. RAD51 and γH2AX foci were counted in at least 100 randomly selected cells per sample. **d** Comet assay-based assessment of DNA DSB HCT116 cells subjected to different treatments as indicated at 48 hpi and 5 days post irradiation (dpi). The extent of DNA damage was measured in 100 randomly selected cells per sample. Data from three repeat experiments were included in the statistical analysis

To investigate whether p73 also promotes damage repair in genomic DNA DSBs, we analysed the formation of DNA DSB repair foci, comprising phosphorylated H2AX (γH2AX, an early marker of DNA DSB) and RAD51 (recombinase involved in HR repair), at 12 hpi. The knockdown of p73 significantly decreased the frequencies of RAD51 focus formation and co-localisation of RAD51 with γH2AX foci at 12 hpi, whereas the overexpression of p73 significantly increased the efficiencies (Figs. 3a–c, S4, S5). The results also showed that the knockdown of p73 increased the formation of γH2AX foci, whereas this process was significantly decreased by the overexpression of p73 (Figs. 3a–c, S4). As γH2AX foci represent unrepaired DNA DSBs while RAD51 foci indicate HR repair progression, the results suggest that p73 promotes DNA DSB repair foci formation.

Next, we performed a comet assay to determine the effects of p73 on the accumulation of DNA DSBs upon γ-irradiation. The extent of DNA damage was significantly increased in p73-knockdown cells (~2.7-fold at 2 dpi and 2-fold at 5 dpi), but was significantly decreased in p73 overexpression cells (0.5-fold at 2 dpi and 0.6-fold at 5 dpi) (Fig. 3d). These results demonstrate that p73 promotes genomic DNA DSB repair upon γ-irradiation.

### Knockdown of p73 inhibits cell proliferation by arresting cell cycle at the G2 phase and promoting cell senescence upon γ-irradiation

**Fig. 4:**
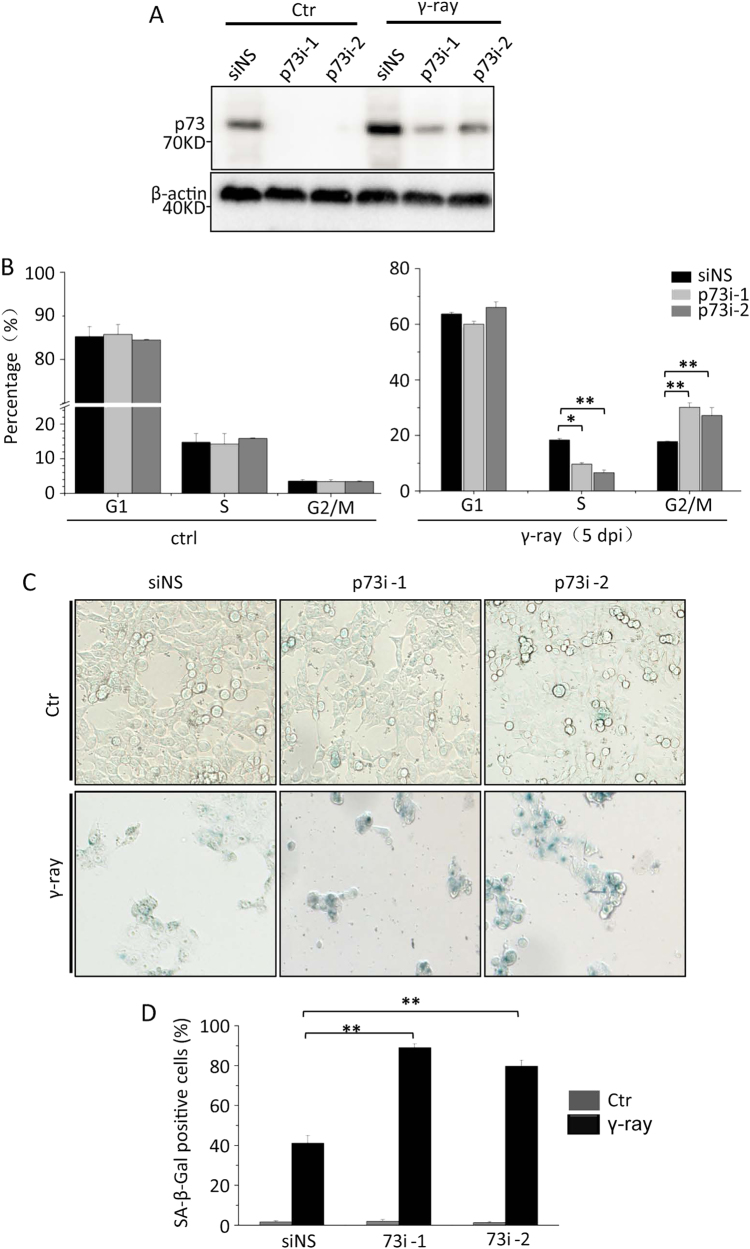
Knockdown of p73 arrests cell growth at the G2 phase of cell cycle and promotes cell senescence upon γ-irradiation. **a** Western blot analysis of p73 in HCT116 cells transfected with siNS, p73i-1 or p73i-2, followed by 10 Gy of γ-ray irradiation. **b** Flow cytometry analysis of the percentages of propidium iodide (PI)-stained cells in different phases of cell cycle. HCT116 cells were transfected with siNS, p73i-1 or p73i-2 siRNA at 5 dpi as indicated. **c**, **d** Senescence-associated β-galactosidase (SA-β-gal) staining was performed to analyse the senescence statuses of HCT116 cells subjected to different treatments as described in Fig. 5b (**c**). Senescent cells in different samples, as shown in Fig. 5c, were subjected to a statistical analysis (**d**)

To study the consequences of increased DNA damage at the cellular level, we performed FACS analysis on cell cycle with PI staining. The results showed that p73 knockdown had no obvious effects on cell cycle in untreated cells at 5 dpi. However, the proportion of cells in the G2 phase increased significantly in irradiated p73-knockdown cells (30.2% in p73i-1 and 27.2% in p73i-2 transfected cells), compared to that in irradiated control cells (17.8%). In contrast, the proportion of cells in the S phase decreased significantly in irradiated p73-knockdown cells (9.7% in p73i-1 and 6.6% in p73i-2 transfected cells), compared to that in irradiated control cells (18.4%) (Figs. 4a, b, S6). However, the irradiated p73 knockdown and control cells did not differ considerably in the proportion of cells in the G1 phase. These results suggest that high-level DNA damage in irradiated p73-knockdown cells causes cell growth arrest at the G2 phase.

The senescence-associated β-galactosidase (SA-β-gal) staining showed that p73 knockdown significantly increased the proportion of positive cells (~89.1% in p73i-1 and 79.7% in p73i-2 transfected cells) at 5 dpi, compared to the irradiated controls (~41.2%) (Fig. 4c, d). Taken together, the loss of function of p73 appeared to increase DNA DSBs upon γ-irradiation, which consequently inhibited cell proliferation by arresting the cell cycle at the G2 phase, leading to cell senescence.

### P73 forms a complex with Δ133p53 upon γ-irradiation

**Fig. 5:**
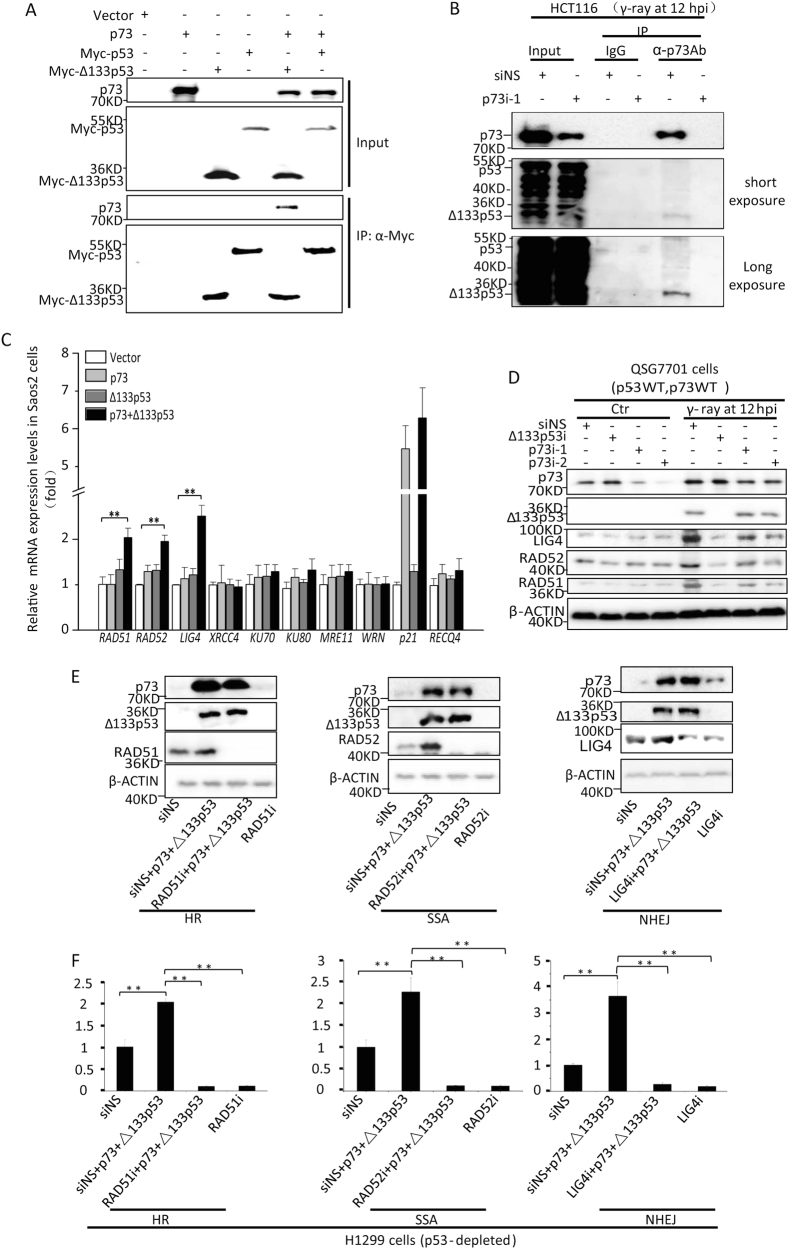
p73 and Δ133p53 form a complex to upregulate the expression of the DNA DSB repair genes *RAD51*, *RAD52* and *LIG4*. **a** Co-immunoprecipitation (IP) analysis of the interaction between p53 or Δ133p53 and p73 under overexpression conditions. 293 T cells were transfected with *CMA-p73*, *CMA-Myc-p53*, *CMA-Myc-Δ133p53*, *CMA-p73* plus *CMA-Myc-p53* or *CMA-p73* plus *CMA-Myc-Δ133p53* plasmids. An anti-Myc antibody was used for the IP. The control contained 10% of input from each sample. **b** Co-IP analysis of the interaction between p53 or Δ133p53 and p73 upon γ-irradiation. HCT116 cells were transfected with siNS or p73i-1, followed with 10 Gy of γ-ray irradiation. The total proteins were sampled at 12 hpi. An anti-p73 antibody was used for IP. IgG was used as a negative control. The control contained 10% of input from each sample. A polyclonal antibody, CM1, was used to detect both p53 and Δ133p53. Middle panel: with a short exposure time; Bottom panel: with a long exposure time. **c** Relative mRNA expression of the listed genes in Saos2 cells overexpressing p73, Δ133p53 or both p73 and Δ133p53 as measured by quantitative real-time (qRT)-PCR at 12 hpi. Gene expression was normalised against *β-ACTIN* and expressed as the fold change compared to the vector transfection control. **d** Western blot analysis of proteins in QSG7701 cells subjected to different treatments as indicated. Protein extracts were analysed via western blotting with appropriate antibodies. **e**,** f** The roles of RAD51, RAD52 and LIG4 in the DNA DSB repair pathways, in the context of p73 and Δ133p53 overexpression. Under different conditions, specific siRNAs were used to knockdown *RAD51*, *LIG4* or *RAD52* in H1299 cells overexpressing p73 and Δ133p53 together with a HR, NHEJ or SSA reporter construct. Western blot analysis of p73, Δ133p53, RAD51, RAD52 and LIG4 from H1299 cells transfected with different reagents as indicated (**e**). The average repair frequencies were measured using a qPCR analysis of the repaired assay constructs from three repeat experiments at 24 hpt (**f**)

According to several studies, p73 does not form a complex with full-length p53 [38–40]. However, p73 was found to interact with ectopically expressed Δ133p53 [41]. Our co-immunoprecipitation (Co-IP) results in 293T cells also showed that Myc-Δ133p53, but not full-length p53, formed a complex with p73 (Fig. 5a). To ascertain whether endogenous p73 and Δ133p53 could form a complex, we transfected HCT116 cells with siNS or p73i-1 followed by 10 Gy of γ-irradiation, and used a p73 antibody for immunoprecipitation. The western blot revealed that Δ133p53, but not full-length p53, co-immunoprecipitated with p73 at 12 hpi (Fig. 5b). Therefore, our data demonstrate that the interaction of Δ133p53 and p73 proteins was not limited to the condition of overexpression, but also occurred in vivo when both proteins were upregulated following γ-irradiation.

### p73 coordinates with Δ133p53 to promote the expression of key DNA DSB repair genes

Next, we used Saos2 cells to analyse the expression of DSB repair and p53-response genes in the context of p73 and Δ133p53. As expected, p73 overexpression significantly upregulated the expression of a p53-responsive gene, *p21* (a cell cycle inhibitor), whereas co-expression with Δ133p53 did not influence p73 to transcribe *p21*. In contrast, the overexpression of either p73 or Δ133p53 alone did not affect the expression of any of the nine DSB repair genes (*RAD51*, *RAD52*, *LIG4*, *XRCC4*, *KU70*, *KU80*, *MRE11*, *WRN* and *RECQ4*). However, the co-expression of p73 and Δ133p53 significantly upregulated the expression of *RAD51*, *RAD52* and *LIG4*, consistent with the finding that Δ133p53 and p73 worked together to enhance the efficiencies of the three DNA DSB repair pathways (Fig. 5c).

We confirmed the role of p73 in the expression of these three repair-related genes in two cell lines: the HCT116 and the normal liver epithelial cell line QSG-7701 (p53^+/+^;p73^+/+^). Protein analysis showed that RAD51, LIG4 and RAD52 were all upregulated in both cell lines at 12 hpi, but were downregulated by the knockdown of either Δ133p53 or p73 (Fig. 5d, Figure S7).

Previous studies have demonstrated that *RAD51*, *LIG4* and *RAD52* are required for HR, NHEJ and SSA repairs, respectively [42–46]. Accordingly, we investigated the roles of these three proteins in the DNA DSB repair pathways in the context of p73. In consistent with the results of RT-PCR and the knockdown of p73 and Δ133p53 in response to γ-irradiation, the western blots showed that the co-expression of p73 and Δ133p53 increased the levels of the three repair-related proteins in H1299 cells (Fig. 5e). Although the co-expression of p73 and Δ133p53 increased the efficiencies of the three DNA DSB repair pathways, the knockdown of *RAD51*, *LIG4* or *RAD52* significantly reduced the repair efficiency of the corresponding pathway to similar levels in cells with and without p73 and Δ133p53 over-expression (Fig. 5e, f). In contrast, the overexpression of *RAD51*, *LIG*4 or *RAD52* significantly increased the repair efficiency of the corresponding pathway to similar levels in HCT116 cells, regardless of the p73 expression status (Figure S8). Together, these data suggest that p73 requires the upregulation of these three repair genes to promote DNA DSB repair.

### Δ133p53 and p73 join together to bind to the promoters of *RAD51*, *LIG4* and *RAD52*

**Fig. 6:**
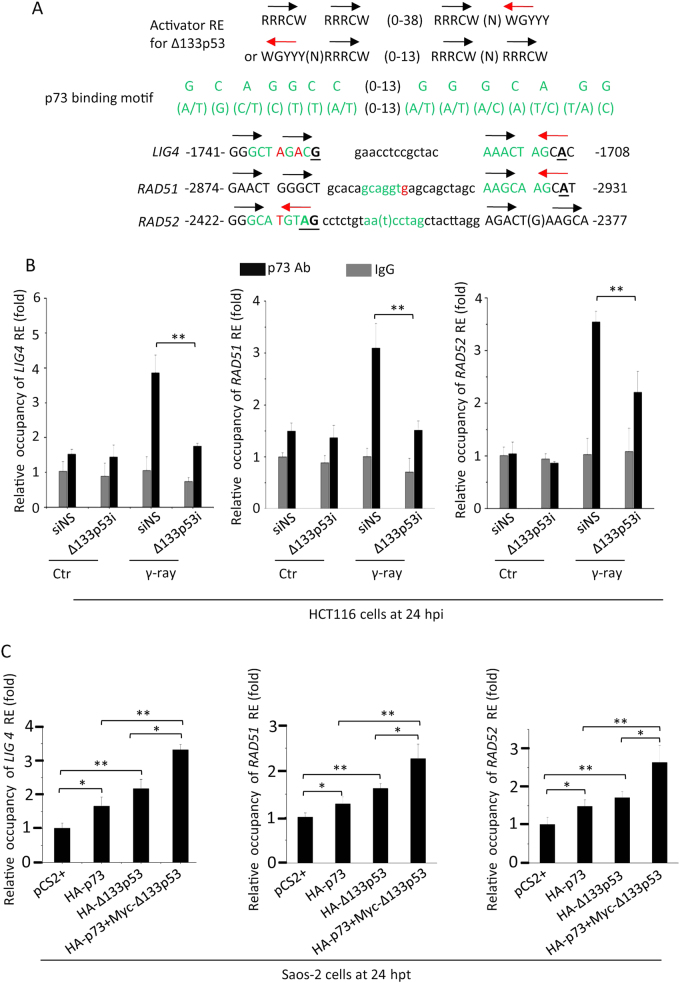
Δ133p53 and p73 act synergistically to bind to the promoters of *RAD51*, *RAD52* and *LIG4*. **a** New types of p53 REs relevant for Δ133p53 transcription activation in the promoters of *RAD51*, *RAD52* and *LIG4*. The black and red arrows correspond to the orientations of the quarter sites of p53 (RE). R = A or G, W = A or T, Y = C or T. The numbers indicate the positions of p53 REs in the three gene promoters. The p53 REs are indicated by uppercase letters. Mismatch nucleotides in p53 REs are underlined. The p73 REs are in green letters. Mismatch nucleotides in p73 REs are labelled with red letters. **b** Chromatin immunoprecipitation (IP) of p53 and p73 REs in the *RAD51*, *RAD52* and *LIG4* promoters in HCT116 cells at 24 hpi. HCT116 cells were transfected with non-specific siRNA (siNS) or Δ133p53 interference (Δ133p53i), followed with 10 Gy of γ-ray irradiation. An N-terminal p73 antibody was used for protein–DNA complex IP. IgG was used as a non-specific binding control. Specific primer pairs were designed to amplify the corresponding REs. DNA was normalised to *β-ACTIN* (negative control primers). The results are presented as relative occupancies of the different REs. Statistics were obtained from three repeat experiments. **c** IP of p53 and p73 REs in the *RAD51*, *RAD52* and *LIG4* promoters in Saos-2 cells transfected with *HA-p73*, *HA-Δ133p53* or co-transfected *HA-p73* with *Myc-Δ133p53*. The transfected cells cells were sampled at 24 hpt. A HA antibody was used for protein–DNA complex IP. The ChIP assay was performed as described in **b**

Our previous study demonstrated that there is a Δ133p53 RE in the promoters of *RAD51*, *LIG4* and *RAD52* [3]. The Δ133p53-activated RE contains two pairs of pentamers, including one pair arranged end-to-head, 5′-RRRC(A/T)(N)RRRC(A/T)-3′, and another pair arranged end-to-end, 5′-RRRC(A/T)(A/T)GYYY-3′ (Fig. 6a). To investigate whether endogenous p73 requires Δ133p53 to bind to the three repair gene promoters upon DSBs, we performed a chromatin immune-precipitation (ChIP) assay with a p73 antibody. The ChIP assay revealed the significant enrichment of p73 in REs within the three repair gene promoters at 24 hpi, whereas the knockdown of Δ133p53 significantly decreased the enrichment of p73 at these promoters (Fig. 6b).

Interestingly, we also found a p73 binding motif [47, 48] within the regions of Δ133p53 RE of the three repair gene’s promoters (Fig. 6a). To analyse whether p73 alone binds to REs in these three repair gene promoters, we performed another ChIP assay in Saos2 cells with over-expression of HA-p73, HA-Δ113p53 or HA-p73 plus Myc-Δ133p53. The assay showed that the occupancies of HA-tagged proteins at the REs of three repair genes were increased in a small degree in the cells expressed either HA-p73 (1.28, 1.47 and 1.65-fold at each promoter) or HA-Δ133p53 (1.62, 1.7 and 2.1-fold) alone, compared to those in the control cells (Fig. 6c). However, the enrichments were significantly increased in the cells co-expressed both HA-p73 and Myc-Δ133p53 (2.27, 2.5 and 3.3-fold), compared to those in cells expressed with either HA-p73 or HA-Δ133p53 alone (Fig. 6c). These results were consistent with that both of p73 and Δ133p53 are required to upregulate the expression of the three repair genes. Taking together, p73 and Δ133p53 act synergistically to bind to the promoters of the three repair genes.

### Mutations in the *Δ133p53* promoter from human cancer tissues attenuate the activation of *Δ133p53* in response to DNA damage

**Fig. 7:**
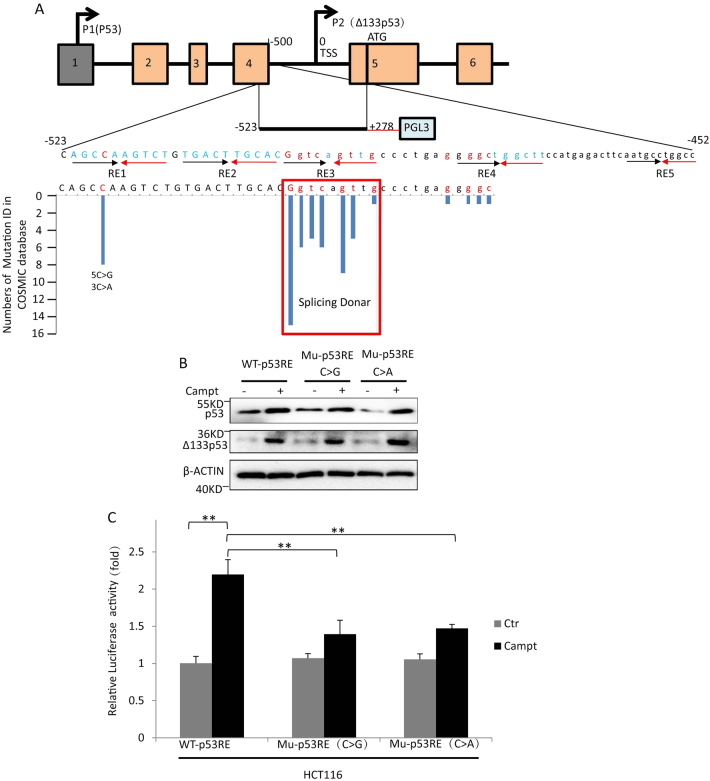
Mutations in the p53 REs of Δ133p53 promoter from cancer tissues attenuate the activation of Δ133p53 in response to DNA damage. **a** Diagram of mutations in the five p53 consensus decamers of Δ133p53 promoter from the COSMIC database. The top panel: the promoter of Δ133p53; P1: the transcription start site for full-length p53; P2: the transcription start site for Δ133p53; ATG: the start codon of Δ133p53; numbers: relative positions to Δ133p53 transcription start site; the middle black line: the region was cloned as the Δ133p53 promoter; black and red arrows: the orientations of the quarter sites of p53 REs; Letters in uppercase: exon-4; Letters in lowercase: intron-4; Letters in red: mutated residues. The bottom panel: total number of mutation IDs in each residue from the COSMIC data base. Red square: putative splicing donor site of intron-4. **b** Western blot analysis of p53 and Δ133p53 proteins in HCT116 cells subjected to different treatments as indicated. HCT116 cells were transfected with the Δ133p53 promoter with WT p53RE of pi3i4-luc construct (WT p53RE), or the mutant promoters with C > G or >A of the reporter plasmids (Mutant p53RE-C > G or Mutant p53RE-C > G), followed with a treatment of camptothecin (Campt). The protein was sampled at 12 hpt. **c** Relative luciferase activity in cells transfected with three constructs and treated with or without Campt. The experiments were performed as described in B. The dual luciferase assay was analysed in duplicate and all results shown are the average of three repeats

To investigate whether the loss of Δ133p53 function promotes tumorigenesis, we searched for mutations in the COSMIC, a public database. As the entire Δ133p53 coding sequence completely overlaps the full-length p53, mutations in the former coding region also cause changes in the latter protein. Therefore, we only searched for mutations in Δ133p53 promoter, of which locates at the junction of exon-4 and intron-4 and contains five putative p53 consensus decamers required for the activation of Δ133p53 transcription. We identified several somatic mutations in p53 decamers from different types of cancer samples (Table S1). Among the five decamers, the third decamer had the highest mutation rate (different mutations in 48 Mutation ID) (Fig. 7a, Table S1). Notably, this decamer is also the intron-4 splicing donor site, and mutations in this motif might affect the splicing of full-length p53. Thus, we focused on the mutations in the remaining four decamers. Five mutations in the four residues of these four decamers were identified in 18 specimens from different cancer tissues (Fig. 7a, Table S2). Interestingly, one of the mutated residues with high mutation rate (in ten specimens) was located in exon-4, and although mutations of C > A or >G do not change the resulting Thr amino acid (ACC > ACG or >ACA) in the full-length p53 protein, the C is a very important consensus residue in the p53 RE (Fig. 7a and Table S2). Next, we cloned the Δ133p53 promoter containing five p53 consensus decamers into a luciferase reporter construct (Fig. 7a) [8]. Two single-residue mutations (C > G or >A) were generated from the construct. Western blots showed that both of endogenous full-length p53 and Δ133p53 was upregulated to a similar level by the treatment of camptothecin (Campt), a DNA damage drug, in different transfected HCT116 cells (Fig. 7b). The luciferase activity assay showed that the promoter activity had no much difference between different untreated transfected cells. The treatment of Campt significantly increased the promoter activity in the WT construct (2.1-fold). However, the increase of promoter activity was significantly attenuated by both of the mutations (1.4-fold in C > G, 1.5-fold in C > A) (Fig. 7c). The data suggest that reduce of Δ133p53 activation in response to DNA damage may be related to tumorigenesis.

## Discussion

The p53 gene family contains three members, p53, p63 and p73, each of which encodes a variety of isoforms that are transcribed from two different promoters or result from alternative splicing [49–52]. Δ133p53, an N-terminal truncated p53 isoform, is strongly induced by γ-irradiation to promote DNA DSB repair via enhancing the transcription of three repair genes, *RAD51*, *RAD52* and *LIG4*, in a p53-independent manner, leading to questions regarding how Δ133p53 lacking the transactivation domain, induces the transcription of these repair genes [19]. In this study, we revealed that the expression of both p73 and Δ133p53 proteins increased to the highest level at 24 hpi (Fig. 1a) and that p73 form a complex with Δ133p53, but not p53, after γ-irradiation (Fig. 5b). Using EGFP-repairing-aided visual-plus-quantitative analysis reporter systems, comet assays and repair focal analyses, we revealed that Δ133p53 coordinates with p73 to promote all three DNA DSB repair pathways by increasing the expression of *RAD51*, *RAD52* and *LIG4* (Figs. 2, 3 and 5). Furthermore, p73 requires Δ133p53 to transcribe the expression of these three key DNA DSB repair genes, as Δ133p53 promotes the binding of p73 to the gene promoters (Fig. 6). Accordingly, we demonstrated that p73 and Δ133p53 work together to ensure genomic integrity upon DNA DSBs.

p73 can be activated upon DNA damage either via protein phosphorylation by c-Ab1 tyrosine kinase [31], or via upregulation of its mRNA expression by the transcription factor E2F1 [34]. However, it remains unclear why p73 is activated via two mechanisms in response to DNA damage. In this report, we found that upon γ-irradiation, p73 protein expression peaked twice, at 4 and 24 hpi (Fig. 1a, b). Our qRT-PCR analysis demonstrated an upregulation of p73 mRNA at 12 hpi, but not before 4 hpi (Fig. 1c, Figure S2). Furthermore, c-Abl knockdown reduced the accumulation of p73 protein at 4 hpi, but not at 24 hpi (Fig. 1e). The depletion of p73 reduced early-stage (before 6 hpi), but not later-stage apoptotic activity (24 hpi) (Figure S3) and increased DNA DSB accumulation at 2 dpi and 5 dpi (Fig. 3d). Taken together, these results suggest that the initial p73 protein peak is caused by protein stabilisation with the aim of promoting apoptosis in severely DNA-damaged cells, whereas the second p73 protein peak is expressed driven by mRNA transcription to increase DNA damage repair in less damaged cells. The consequent increase in DNA DSBs in p73-knockdown cells led to cell cycle arrest in the G2 phase and, ultimately, cell senescence (Fig. 4), consistent with the results of a previous study involving Δ133p53 knockdown [3, 53]. Therefore, the loss function of p73 in the context of DNA DSB repair may be a major cause of the spontaneous and carcinogen-induced tumorigenesis observed in p73-knockout mice [27].

The expression of Δ133p53 has been found to be elevated in a variety of tumours such as: breast cancer, renal cell carcinoma, ovarian cancer, intrahepatic cholangiocarcinoma, colon cancer and lung carcinoma [6, 16, 54–59]. Depletion of Δ133p53 inhibited angiogenesis and growth of glioblastoma [18]. All of these studies demonstrated that the overexpression of Δ133p53 is associated with carcinogenesis due to its role in anti-apoptosis and promoting angiogenesis. Interestingly, a previous analysis of germline p53 mutations in breast cancer revealed that the Li-Fraumeni and Li-Fraumeni-like syndromes are closely related with the loss of the initiation codon of the Δ133p53 isoforms, which suggests that the Δ133p53 isoforms are required for the genetic stability in germline cells [60]. From the COSMIC cancer database, we found there are a large number of somatic mutations in the promoter region of Δ133p53 (Fig. 7a, Table S1, S2). The mutations in one of residues locating in exon-4 do not change the codon of p53 protein, but attenuated the activation of Δ133p53 in response to the DNA damage (Fig. 7). The data suggest that the loss of Δ133p53 function might be also associated with tumorigenesis due to its role in DNA damage repair.

## Materials and methods

### Cell culture

H1299 (TCHu160), Saos-2, 293T/7 (HEK 293T/7) and HCT116 cells were purchased from the Cell Bank of the Chinese Academy of Sciences (Shanghai, China). Cell transfections were performed using PolyJet™ transfection reagent (SignaGen Laboratories, Rockville, MD, USA) for plasmids and Lipofectamine^TM^ 2000 transfection reagents for siRNA (GIBCO, Grand Island, NY, USA).

### qRT-PCR

For qRT-PCR, total RNA was obtained from cells subjected to different treatments, treated with Dnase I and purified via lithium chloride precipitation. M-MLV Reverse Transcriptase (Invitrogen, Carlsbad, CA, USA) was used to synthesise cDNA from total RNA. The qRT-PCRs were performed on a CFX96^TM^ real-time system (Bio-Rad, Hercules, CA, USA), using AceQ qPCR SYBR Green Master Mix (Vazyme, Nanjing, China) according to the manufacturer’s instructions. The relative gene expression levels were normalised to that of *β-ACTIN*. The results of three repeat experiments were used for the statistical analysis, and significant differences were assessed using the independent-samples *T*-test. The primers for p73 isoform amplification were designed as described in previous reports [22, 61–64]. The sequences of primers used in this research are listed in Supplemental Table S3.

### HR, NHEJ and SSA assays

The HR, SSA and NHEJ visual-plus-quantitative assay systems and analytical procedures were constructed and performed as described previously [37]. H1299 and Saos-2 cells were used for the human cell-based HR, SSA and NHEJ assays. Specifically, 1.5 μg of I-*Sce*I-cut (HR), 0.5 μg of I-*Sce*I-cut (NHEJ) or 0.5 μg of I-*Sce*I-cut (SSA) plasmid DNA were co-transfected with 100 nmol p73i-1, 100 nmol p73i-2, 1.5 μg *CMV-Δ133p53*, 100 nmol p73i-1 plus 1.5 μg *CMV-Δ133p53*, 100 nmol p73i-2 and 1.5 μg *CMV-Δ133p53*, 0.5 μg *CMV-p73*, 0.5 μg *CMV-p73* plus 1.5 μg *CMV-Δ133p53* into 10^6^ H1299 cells. Cells were transfected with uncut plasmid as the negative control. The transfected cells were cultivated for 24 h at 37 °C, followed by DNA extraction for qPCR analysis.

Human *p73, RAD51, RAD52* and *LIG4* were amplified using the gene specific primer pair and then cloned into pCS2+ vector. The primer sequences are provided in Supplementary Table S1.

### qPCR

For the quantitative PCR (qPCR) assays, DNA was extracted at 24 h post treatment using a DNA extraction kit according to the manufacturer’s protocol (Aidlab, Beijing, China). The PCR was performed with a Bio-Rad CFX96/C1000 real-time PCR machine. The amounts of transfected DNA were normalised using normalising primers The frequencies of HR, NHEJ and SSA repairs were quantified using the respective pairs of repair primers, as described in our previous study [37].

### Comet assay

For the comet assay, HCT116 cells were transfected with 100 nmol of a mixture of p73i-1 and -2 or with 0.5 μg *CMV-p73*, followed by γ-irradiation. At 48 hpi, the irradiated cells were fixed in 70% ethanol and subjected to cell dissociation in ice-cold phosphate-buffered saline (PBS) containing 20 mM EDTA (without Mg^2+^ and Ca^2+^). The assay was performed using a OxiSelect^TM^ comet assay kit (Cell Biolabs Inc., San Diego, CA, USA) according to the manufacturer’s recommendations. Embedded cells were treated with lysis buffer at pH 7 (i.e. non-alkaline) to release the double-stranded DNA. For data processing, each comet picture was measured using ImageJ software, version 1.45 (National Institutes of Health, Bethesda, MD, USA), and the extent of damage in individual cells was calculated as described previously [3].

### Flow cytometry analysis

Transfected cells were treated with 10 Gy of irradiation 24 h prior to irradiation. To assay apoptosis at 0, 4, 6 and 24 hpi, the cells were digested with trypsin, washed three times with chilled PBS and stained with PI and Annexin V (Annexin V Apoptosis Detection Kit, Beyotime, Jiangsu, China). The cells were subsequently subjected to analysis on a FACSCalibur flow cytometer (BD Biosciences, San Jose, CA, USA).

For the cell cycle analysis, 24 h after transfection, cells were treated with 10 Gy of irradiation. As described in previous studies, apoptosis decreased to the basal level at 36 hpi [3]. We washed away apoptotic cells at 2 dpi and replaced the culture medium to allow the remaining cells to grow under normal conditions. At 5 dpi, the cells were fixed with 70% ethanol, stained with PI and subjected to flow cytometry analysis. A minimum of 5 × 10^4^ cells per sample were analysed.

### SA-β-gal staining

To perform SA-β-gal staining, HCT116 cells were transfected with siRNAs and exposed to γ-irradiation, as described for the apoptosis and cell cycle assay experiments. At 48 hpi, the irradiated cells were fixed in 4% PFA and subjected to SA-β-gal staining using the Cell Senescence SA-β-Gal Staining Kit (Beyotime, Jiangsu, China). Three repeat experiments were included in the statistical analysis.

### Western blot, Co-IP and immunofluorescence staining

Western blotting was performed as described previously [19]. For the western blot analysis, A p73 monoclonal antibody (#4A4, IMGENEX/Novus Biologicals, Littleton, CO, USA) was used to detect p73. A N-terminal specific p53 monoclonal antibody (#DO-1, Santa Cruz Biotechnology,USA) was used to detect full length p53. A rabbit polyclonal p53 antibody was used to detect full length p53 and Δ133p53 (#NCL-p53-CM1, Novocastra, USA). Rabbit monoclonal antibodies against human RAD51 (#5181-1), RAD52 (#5257-1) and β-Actin (#1854-1) were from Epitomics. A mouse monoclonal antibodies against human LigaseIV (#DR1085) was from Calbiochem.

For the co-IP analysis, transfected cells were cultivated for 48 h at 37 °C, followed by protein extraction. A Myc antibody matrix (Hua An, China) was used for IP. A p73 monoclonal antibody (#4A4, IMGENEX, Littleton, CO, USA) was used to detect p73. A c-Myc monoclonal antibody was used to detect Myc-p53 and Myc-Δ133p53.

For immunofluorescence staining, the cultured cells were plated onto coverslips and placed in six-well plates. To analyse RAD51 (#5181-1, Epitomics, USA) and γH2AX S139 (#05-636, Millipore, USA) focus formation, the cells were collected, washed with hES culture medium and plated on a gelatine-covered Coverglass For Growth (Fisher Scientific, Hampton, NH, USA). After a 6-h incubation, the cells were subjected to immunofluorescent staining as previously described. RAD51 and γH2AX foci were counted in least 100 randomly selected cells per sample.

### ChIP assay

ChIP assays were performed as described previously [3]. For ChIP of endogenous p73, 800 nmol siNS and 800 nmol Δ133p53i were respectively transfected into 10^7^ HCT116 cells. The transfected cells were cultivated for 24 h at 37 °C and then exposed to 10 Gy of γ-irradiation. The chromatin was sheared into 200–500 base-pair (bp) fragments using a Cole–Parmer sonicator equipped with a 2-mm tip. An N-terminal p73 antibody (5B429, Novus Biologicals, Littleton, CO, USA) was used for IP of the sonicated DNA–protein complex solutions. The primers used for qPCR are listed in Supplemental Table S3. The total pulldown DNA was normalised using a pair of non-specific primers for the β-*ACTIN* promoter. Three repeat experiments were included in the statistical analysis.

For ChIP of overexpressed tagged p73 or Δ133p53, 10^6^ Saos-2 cells were transfected with 1.5 μg *HA-Δ133p53*, 0.5 μg *HA-p73* or 0.5 μg *HA-p73* plus 1.5 μg *Myc-Δ133p53*. The protein and DNA complexes were sampled at 24 hpt. HA antibody conjugated agarose beads were used for IP. The detail procedures were described as above.

### Luciferase assay

Luciferase assay was performed as described previously [8]. For luciferase analysis, transfected cells were cultivated for 12 h at 37 °C. For each well, 150 ng of p53 internal promoter construct and 20 ng of the Renilla luciferase reporter plasmid were co-transfected. After transfected 12 h, cells treated with 100 nm Camptothecin or equal volume medium as a negative control for 12 h and then were subjected to the luciferase analysis.The Renilla luciferase reporter plasmid was used as an internal control.

### siRNA

siRNAs and a negative control duplex (non-specific control siRNA siNS) were purchased from Invitrogen. p73i-1, p73i-2, ΔNp73i, Δ133p53i, RAD51i, RAD52i and LIG4i were used as described previously [3, 65] and the sequences of all siRNAs are listed in Table S3.

## Electronic Supplementary material

Supplementary materials
