# Supplementary material for: p73 coordinates with Δ133p53 to promote DNA double-strand break repair
Source: Cell Death Differ. 2018 Mar 6;25(6):1063–79. doi: 10.1038/s41418-018-0085-8 (PMC5988805; doi:10.1038/s41418-018-0085-8)
Supplement: Supplementary file 1 — Supplementary materials [file 41418_2018_85_MOESM1_ESM.pdf]

Supplementary figures and information

Supplementary figures

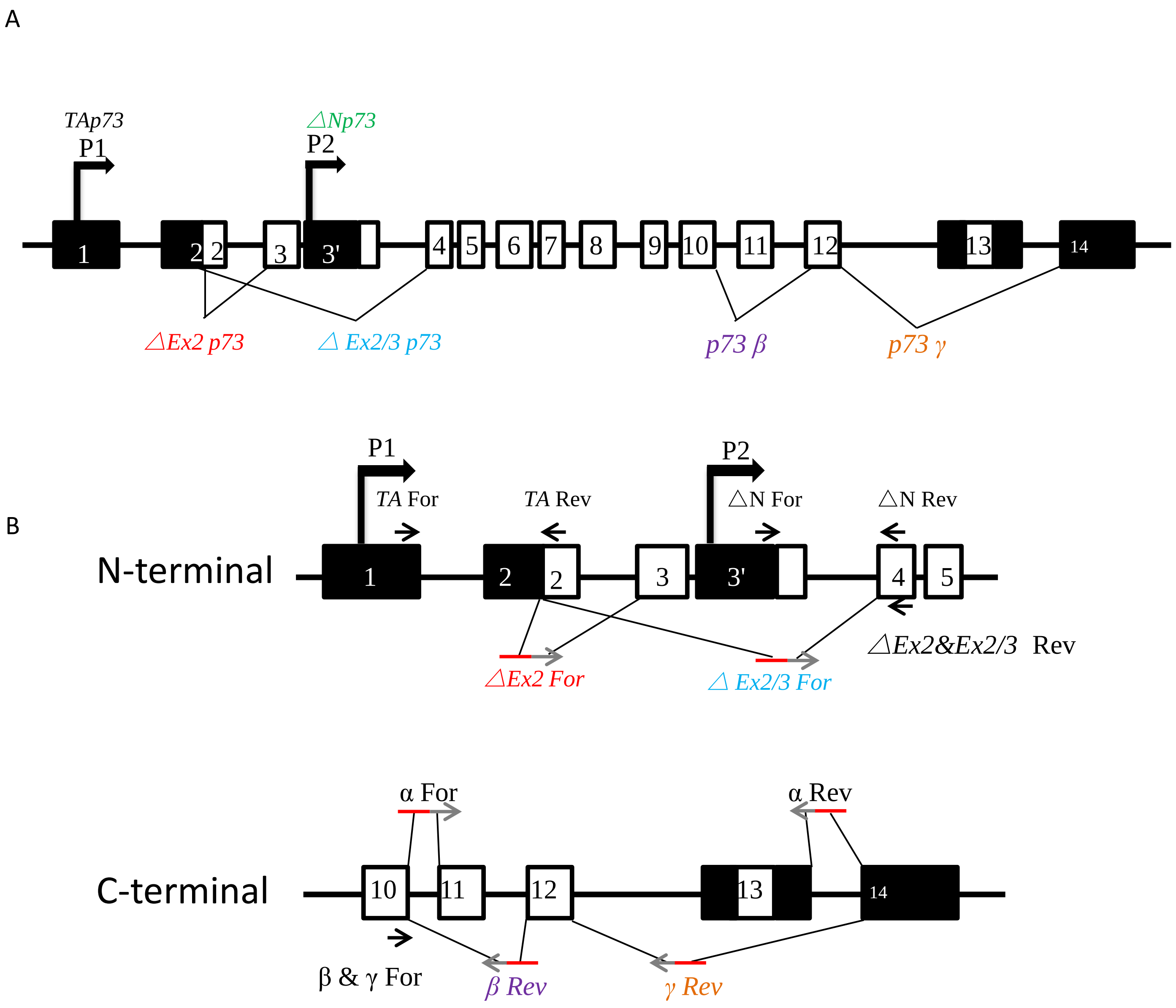
A
TAp73
P1
△Np73
P2
1
2 2
3
3'
4
5
6
7
8
9
10
11
12
13
14
△Ex2 p73
△ Ex2/3 p73
p73 β
p73 γ
B
P1
TA For
TA Rev
P2
△N For
△N Rev
N-terminal
1
2
2
3
3'
4
5
△Ex2&Ex2/3 Rev
△Ex2 For
△ Ex2/3 For
α For
α Rev
C-terminal
10
11
12
13
14
β & γ For
β Rev
γ Rev

Figure S1

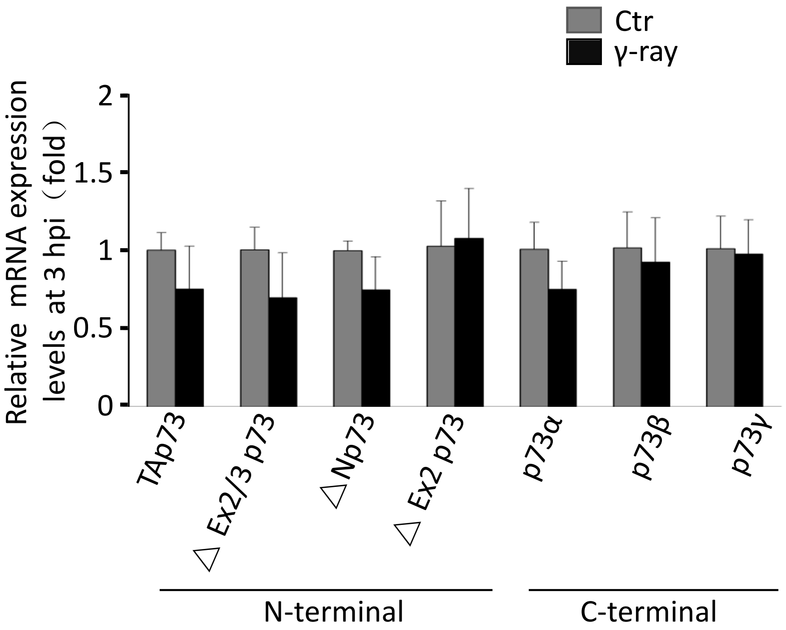
Ctr
γ-ray
Relative mRNA expression levels at 3 hpi（fold）
2
1.5
1
0.5
0
TAp73
△ Ex2/3 p73
△Np73
△ Ex2 p73
p73α
p73β
p73γ
N-terminal
C-terminal

Figure S2

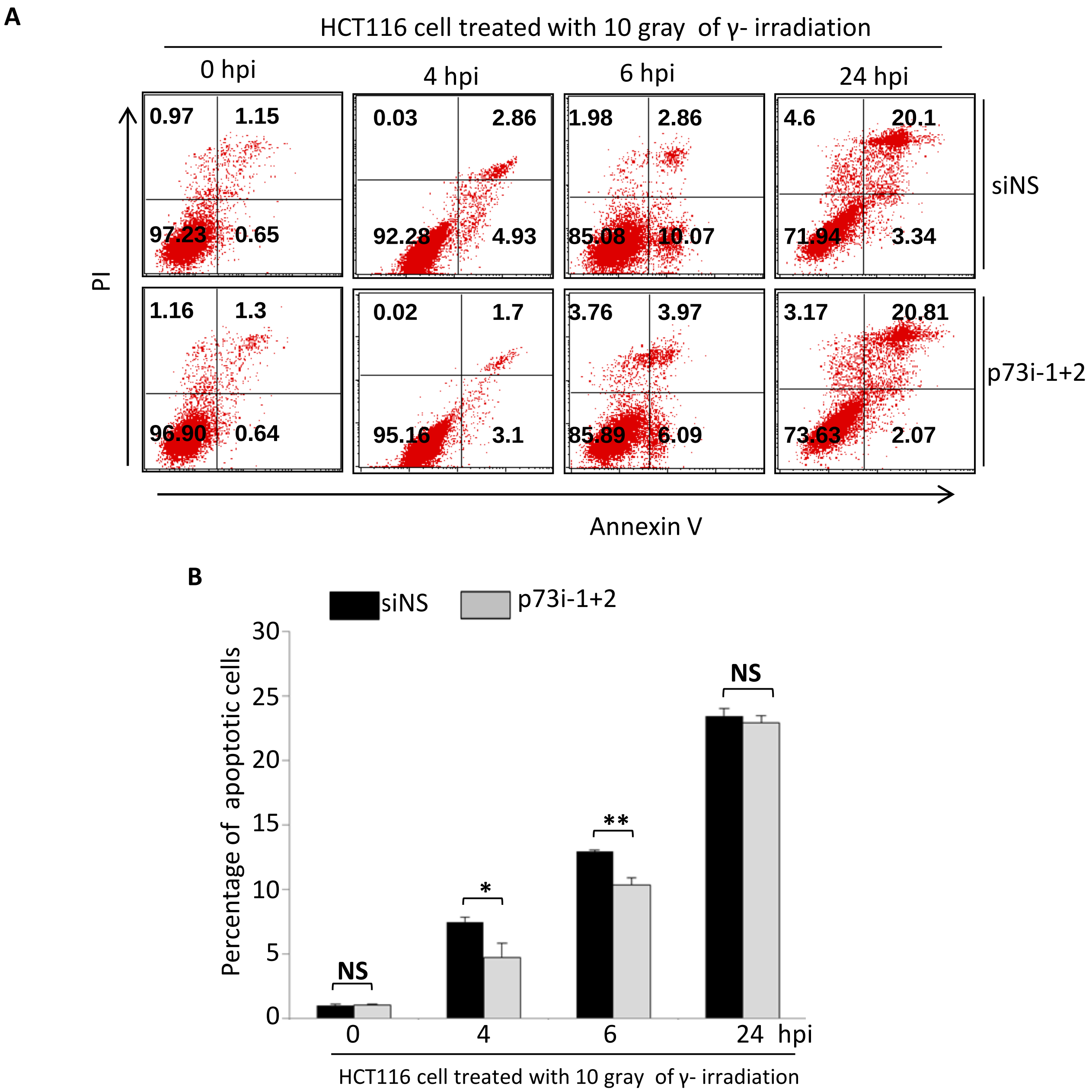

A
HCT116 cell treated with 10 gray of γ- irradiation
0 hpi
4 hpi
6 hpi
24 hpi
0.97
1.15
97.23
0.65
0.03
2.86
92.28
4.93
1.98
2.86
85.08
10.07
4.6
20.1
71.94
3.34
siNS
1.16
1.3
96.90
0.64
0.02
1.7
95.16
3.1
3.76
3.97
85.89
6.09
3.17
20.81
73.63
2.07
p73i-1+2
PI
Annexin V
B
siNS
p73i-1+2
Percentage of apoptotic cells
NS
*
**
NS
0
4
6
24 hpi
HCT116 cell treated with 10 gray of γ- irradiation

Figure S3

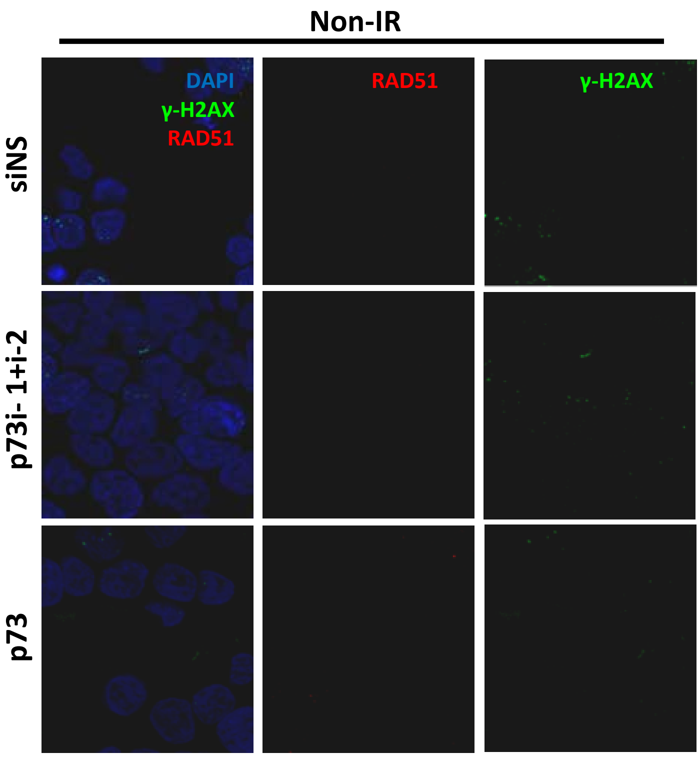

Figure S4

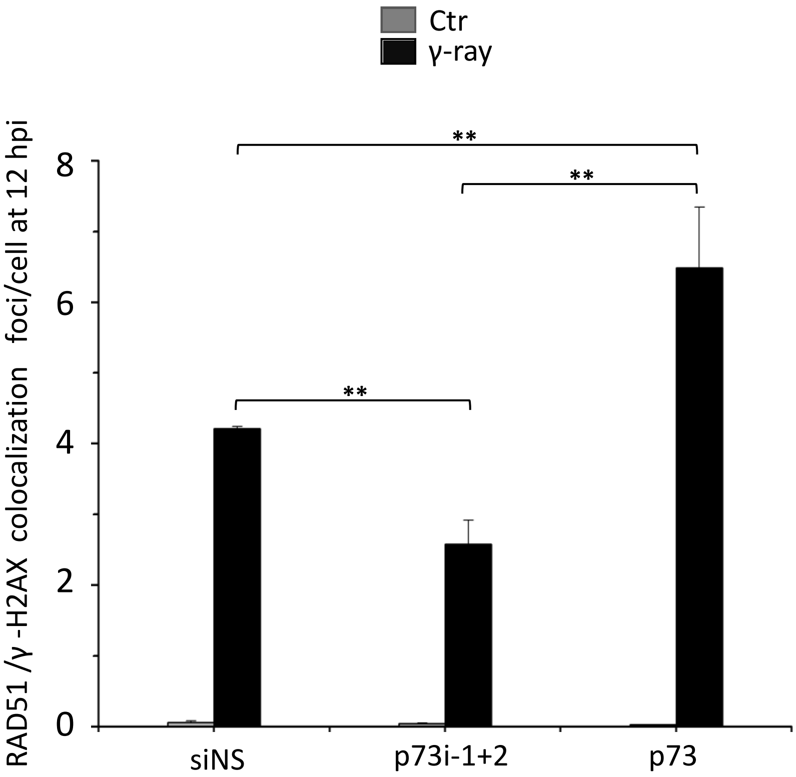

Ctr
γ-ray
RAD51 /γ -H2AX colocalization foci/cell at 12 hpi
8
6
4
2
0
**
**
**
siNS
p73i-1+2
p73

Figure S5

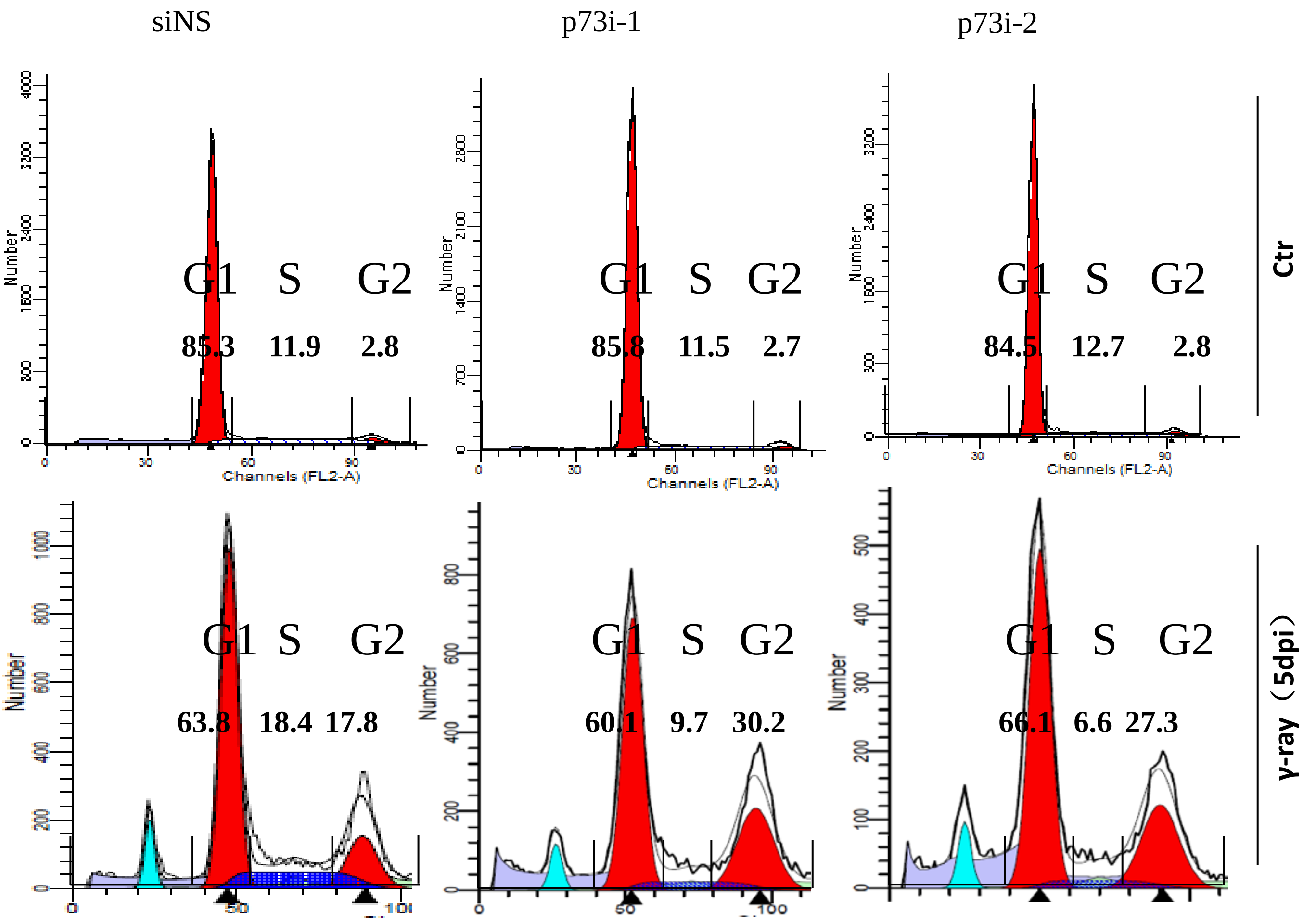

siNS
p73i-1
p73i-2
Ctr
γ-ray（5dpi）
G1 S G2
85.3 11.9 2.8
85.8 11.5 2.7
84.5 12.7 2.8
63.8 18.4 17.8
60.1 9.7 30.2
66.1 6.6 27.3
Number
Channels (FL2-A)

Figure S6

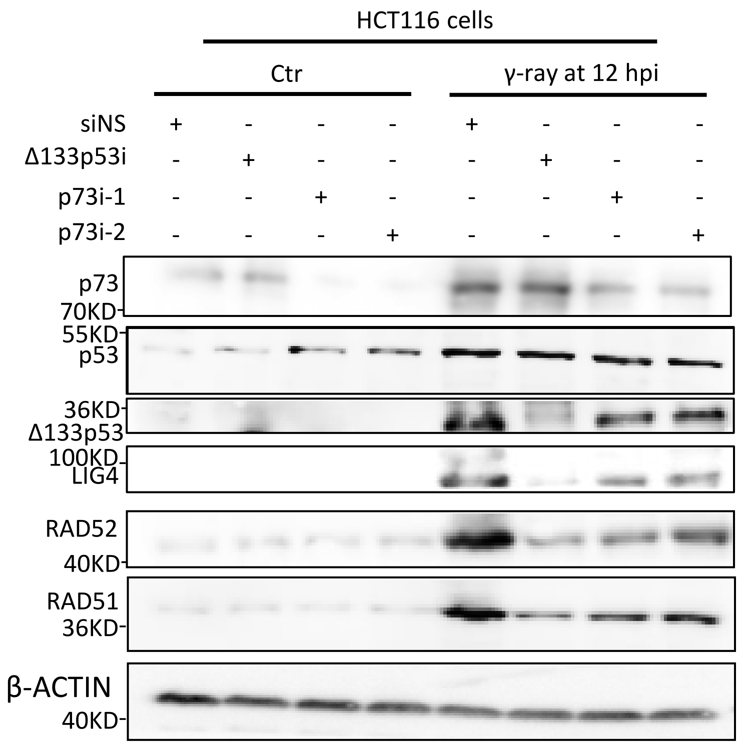
HCT116 cells
Ctr
γ-ray at 12 hpi
siNS
Δ133p53i
p73i-1
p73i-2
p73
70KD
55KD
p53
36KD
Δ133p53
100KD
LIG4
RAD52
40KD
RAD51
36KD
β-ACTIN
40KD

Figure S7

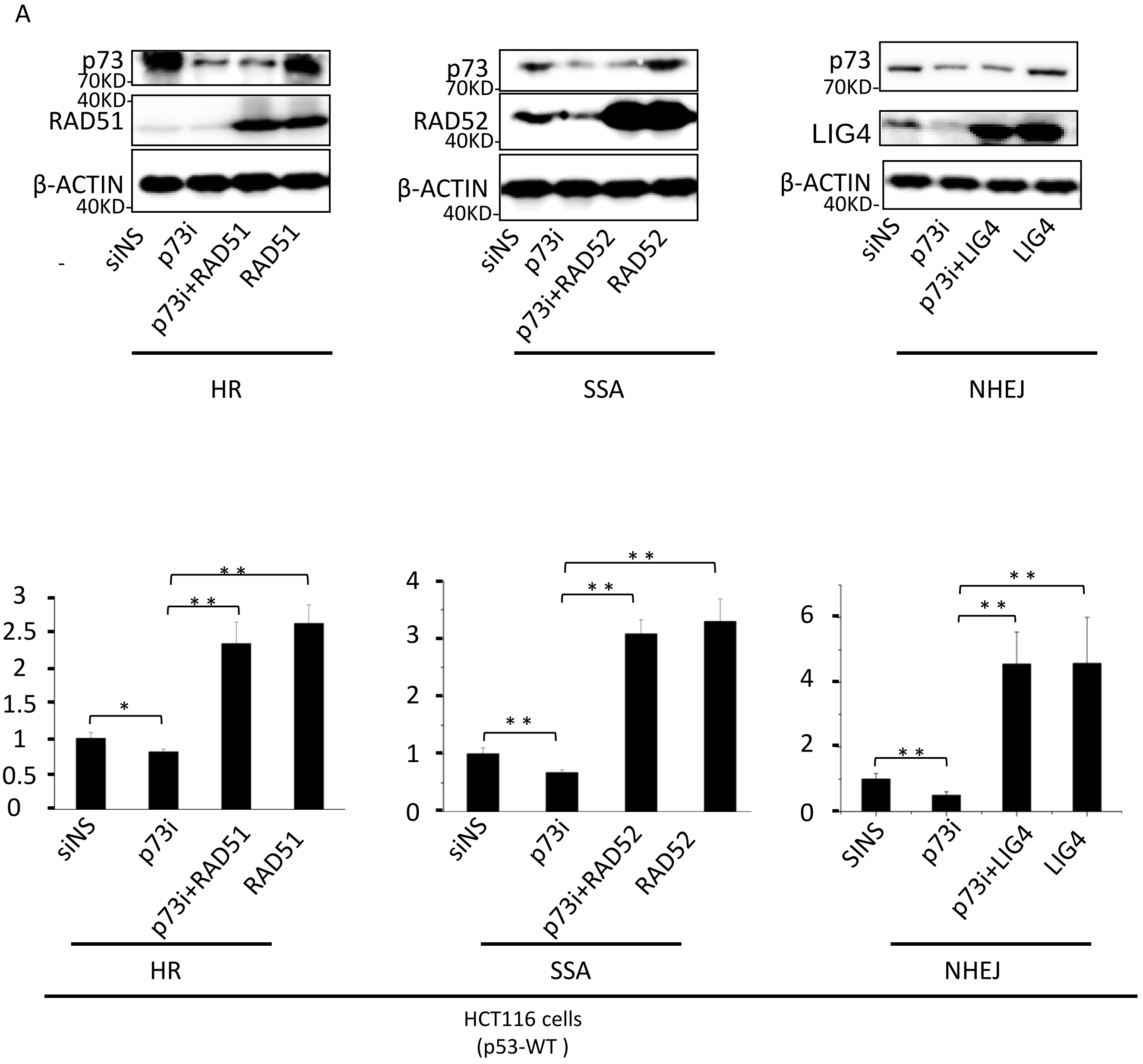
A
p73
70KD
40KD
RAD51
β-ACTIN
40KD
siNS
p73i
p73i+RAD51
RAD51
HR
p73
70KD
RAD52
40KD
β-ACTIN
40KD
siNS
p73i
p73i+RAD52
RAD52
SSA
p73
70KD
LIG4
β-ACTIN
40KD
siNS
p73i
p73i+LIG4
LIG4
NHEJ
B
siNS
p73i
p73i+RAD51
RAD51
HR
siNS
p73i
p73i+RAD52
RAD52
SSA
SINS
p73i
p73i+LIG4
LIG4
NHEJ
HCT116 cells
(p53-WT)

Figure S8

Supplementary information

Supplementary figure legends

Figure S1 Diagram of p73 gene structure. The p73 isoform were identified for the N terminal and the C terminal.The N terminal were divided into TAp73,△Np73, △Ex2p73 and △Ex2&3p73 while the C terminal were divided into α, β, γ. TAp73 , △Ex2p73 and △Ex2&3p73 were transcripted from the P1promoter while △Np73 was transcripted from the P2 promoter.TAP73 was amplificated by TAp73 For and TAp73Rev; △Np73 was amplificated by △Np73 For and △Np73 Rev; △Ex2p73 was amplificated by △Ex2p73 For and △Ex2&3p73 Rev; △Ex2&3p73 was amplificated by △Ex2&3p73 For and △Ex2&3p73 Rev;p73α was amplificated by p73α For and p73 α Rev; p73 β was amplificated by p73 β For and p73 β & γ Rev; p73γ was amplificated by p73γ For and p73 β & γ Rev.The TAp73 forward primer was designed in the exon1 which specifically in the TA isoforms. The △Np73 forward primer was designed in in the exon3' which specifically in the △N isoforms. The △Ex2p73 forward primer was specifically designed in the overlap between exon1 and exon3 while the △Ex2&3p73 forward primer was specifically designed in the overlap between exon1 and exon4.The α Rev primer was specifically designed in the overlap between exon13 and exon14, β Rev primer was specifically designed in the overlap between exon10 and exon12, γ Rev primer was specifically designed in the overlap between exon12 and exon14.

Figure S2 Relative transcript expression of p73 isoforms in HCT116 cells treated with 10 gray γ-irradiation measured by qRT-PCR at 3 hpi. The different isoform transcripts were amplified with a specific pair of primers described in Figure S1. Transcript expression was normalized against *β-ACTIN* and expressed as the fold change compared to untreated control.

Figure S3 p73 promotes apoptosis upon γ-irradiation at early stage, but not at late stage. (A) Representative FACS profiles at 0, 4, 6 and 24 hpi. HCT116 cells were transfected with either siNS or a mixture of p73i-1 and p73i-2, followed by 10 gray of γ-irradiation. The treated cells at different time points as indicated were stained with Propidium Iodide (PI) and Annexin V antibody, and subjected to FACS analysis. (B) Statistic analysis of total apoptotic cells in different samples (including early and late apoptotic cells) as shown from three repeat experiments.

Figure S4 Co-immunostaining of RAD51 (in red) and γH2AX (in green) in HCT116 cells transfected with different reagents as described in Figure 3A, without the treatment of γ-irradiation. The specific monoclonal antibodies were used to determine the RAD51 and γH2AX foci formation as indicated. DAPI was used to stain the nuclear DNA (blue).

Figure S5 Statistical analysis of the average numbers of co-localization of RAD51 and γH2AX foci per cell in different samples, as shown in Figure 3B and Figure S4.

Figure S6 The FACS analysis of the percentage of cells at different cell cycle phases was based on PI staining. The experiment was performed as described in Figure 4B.

Figure S7 Western blot analysis of proteins in HCT116 cells subjected to different treatments as indicated. Protein extracts were analysed via Western blotting with appropriate antibodies.

Figure S8 The roles of RAD51, RAD52 and LIG4 in the DNA DSB repair pathways, in the context of the knockdown of p73. Western blot analysis of p73, RAD51, RAD52 and LIG4 from HCT116 cells transfected with different reagents as indicated (A). The average repair frequencies were measured using a qPCR analysis of the repaired assay constructs from three repeat experiments at 24 hpt (B).

Table S1 The mutation ID in the third p53 consensus decamer from COSMIC database

Mutation ID	DNA Change
COSM44046	chr17:7675986G>A
COSM4969482	chr17:7675988T>G
COSM4969483	chr17:7675988T>G
COSM4969486	chr17:7675988T>G
COSM4969485	chr17:7675988T>G
COSM4969484	chr17:7675988T>G
COSM6222490	chr17:7675989G>A;
COSM6222489	chr17:7675989G>A;
COSM6222493	chr17:7675989G>A;
COSM6222491	chr17:7675989G>A;
COSM6222492	chr17:7675989G>A;
COSM118935	chr17:7675989G>C;
COSM118932	chr17:7675989G>T;
COSM118933	chr17:7675989G>T;
COSM118934	chr17:7675989G>T;
COSM44049	chr17:7675991C>T
COSM46397	chr17:7675992T>A
COSM6148101	chr17:7675992T>A
COSM44436	chr17:7675992T>C
COSM46043	chr17:7675992T>G
COSM6148098	chr17:7675992T>A
COSM1172513	chr17:7675992T>C
COSM1172514	chr17:7675992T>C
COSM6148100	chr17:7675992T>A
COSM3522713	chr17:7675992T>C
COSM6148099	chr17:7675992T>A
COSM3522712	chr17:7675992T>C
COSM45304	chr17:7675993G>A
COSM45910	chr17:7675993G>C
COSM127204	chr17:7675993G>T
COSM437620	chr17:7675993G>A
COSM3970384	chr17:7675993G>C
COSM127205	chr17:7675993G>T
COSM43904	chr17:7675994G>A
COSM292894	chr17:7675994G>A
COSM292893	chr17:7675994G>A
COSM3403299	chr17:7675994G>A
COSM2744976	chr17:7675994G>A
COSM46152	chr17:7675994G>C
COSM437621	chr17:7675994G>C

COSM437622	chr17:7675994G>C
COSM4070058	chr17:7675994G>C
COSM4070057	chr17:7675994G>C
COSM45940	chr17:7675994G>T
COSM381995	chr17:7675994G>T
COSM381996	chr17:7675994G>T
COSM3388231	chr17:7675994G>T
COSM1638003	chr17:7675994G>T

TableS2 Information of Mutations in the remaining four p53 consensus decamers

DNA Change	Mutation ID	Cancer type	Number of specimens	Total Number of cancer tissues
chr17:7676012C>G	COSM4871547	Endometrium	1	3722
	COSM4871546	Endometrium	1	3722
	COSM984974	Endometrium	1	3722
	COSM46304	Endometrium	1	3722
		Urinary tract	1	7691
	COSM984972	Endometrium	1	3722
chr17:7676012C>A	COSM122107	Upper aerodigestive tract	1	4480
	COSM122108	Upper aerodigestive tract	1	4480
	COSM43668	Upper aerodigestive tract	1	4480
		Oesophagus	1	3070
chr17:7675977G>A	COSM44985	Haematopoietic and lymphoid	4	109459
chr17:7675976G>A	COSM44034	Haematopoietic and lymphoid	3	109459

chr17:7675975C>T	COSM45007	Haematopoietic and lymphoid	1	109459

Table S3. PCR Primers and siRNA sequences

**Primers for quantifying frequencies of HR, SSA and NHEJ**	
Normalizing forward primer (Norm-F)	ATCATGGCCGACAAGCAGAAGAACG
Normalizing reverse primer (Norm-R)	CGGCGGCGGTCACGAACTCC
HR and SSA repair forward primer (HR and SSA-Rep-F)	TGACCACCCTGACCTACG
Repair reverse primer (Rep-R):	CACCTTGATGCCGTTCTTCTGC
NHEJ repair forward primer (NHEJ-Rep-F-1)	TCGGAGCAAGCTTGATTTAGGTGA
**Primers for over-expression constructs**	
HA-hup73-ClaI-For	CCATCGATACCATGGAGTACCCATACGACGTACCA GATTACGCTCATGCCCAGTCCACCGCCACCTC
Hup73-XhoI-Rev	CGGTCTCGAGTCAGTGGATCTCGGCCTCCGTGA
Hup73-ClaI-For	GCCAATCGATATGGCCCAGTCCACCGCCACCT
**Primers for qRT-PCR in Hup73 isoforms**	
TA-p73 For	GGCTGCGACGGCTGCAGAGC
TA-p73 Rev	GCTCAGCAGATTGAACTGGGCCATG
ΔN-p73 For	CAAACGGCCCGCATGTTCCC
ΔN-p73 Rev	GAACTGGGCCGTGGCGAG
ΔEx2-p73 For	GGCTGCGACGGCTGCAGGGA
ΔEx2/3-p73 For	GGCTGCGACGGCTGCAGGCC
ΔEx2&ΔEx2/3-p73 Rev	CAGGCGCCGGCGACATGG
Hu-p73α For	AGTCACCTACAGCCCCCGTCCT
Hu-p73α Rev	ATGGTCAGGTTCTGCAGGTGGT
Hu-p73β&γ For	AGTCACCTACAGCCCCCGTCCT
Hu-p73β Rev	TGGACTGGGCGCTGTGGCTGCT
Hu-p73γ Rev	TGGGCGAGAGGACCGGCCCGT
Hu β-ACTIN For	TGGTGGGCATGGGTCAGAAGGAT
Hu β-ACTIN Rev	CCAGAGGCGTACAGGGATAGCAC
**Primers for qRT-PCR in DSB genes expression**	
Hu RAD51 For	ACAGTGCCACCGCCCTTTACA
Hu Rad51 Rev	GGTTTCCCCTCTTCCTTTCCTCA

Hu RAD52 For	TGGCGGCGGCTCAGTGTTATG
Hu RAD52 Rev	ATTCTGCTGCGTGATGGAGTGTGC
Hu LIG 4 For	TAGGGGGAAGGCATCTGGTAAG
Hu LIG 4 Rev	GTGTCTGGGCCTGGATTTTGTA
Hu p21 For	GGAAGACCATGTGGACCTGT
Hu p21 Rev	GGATTAGGGCTTCCTCTTGG
Hu XRCC4 For	TCTTGGGAGAAAACACTGGAA
Hu XRCC4 Rev	TCCTGCTCCTGACAACAATG
Hu Ku70 For	GGGACAAAAACGTTTCCAAG
Hu Ku70 Rev	CCAGGTTTCTTCAGGTGCAT
Hu Ku80 For	ATCCCCATTTGAACAAGCA
Hu Ku80 Rev	CAATGTCCTCCAGCAAATC
Hu MRE11 For	GACTTGCCCAGGAAAATGAA
Hu MRE11 Rev	TGTTGAGGTTGCCATCTTGA
Hu WRN For	GGCTGAAGAAGACCTGTTGG
Hu WRN Rev	GCACATTCATCCATTCAGGA
Hu RECQ4 For	AGTCCCTGTGCTACCAGCTC
Hu RECQ4 Rev	ACAGATTCCCGTTGCTTCC
Hu β-ACTIN For	TGGTGGGCATGGGTCAGAAGGAT
Hu β-ACTIN Rev	CCAGAGGCGTACAGGGATAGCAC
**Primers for qPCR in ChIP assay**	
β-ACTIN-ChIP For	GGACTTCGAGCAAGAGATGG
β-ACTIN-ChIP Rev	AGGAAGGAAGGCTGGAAGAG
p53exon5-ChIP For	CTGCCCTCAACAAGATGTTT
p53exon5-ChIP Rev	CTCCGTCATGTGCTGTGACT
LIG 4-ChIP For	CCTAAGATGGGCAAGTGGAG
LIG 4-ChIP Rev	CAGTGGGCCCTGATAAAACA
RAD51-ChIP For	TAATTCAGAGGACCCCAACG
RAD51-ChIP Rev	TGATCTGACAGGAGGCAGAG
RAD52-ChIP For	ACCTGCAGTTCCAGCACTTT
RAD52-ChIP Rev	GACAGCACCTCACTCTGCTG
**The siRNA sequence *sense（5'-3'）**	
TAp73(exon3) i-1	AACGGAUUCCAGCAUGGACGU
TAp73 i-2	UCUGCUGAGCAGCACCAUG
ΔNp73(exon3B to 4) i	AACCUCGCCACGGCCCAGUUC
▵133p53 i	UGUUCACUUGUGCCCUGACUUUCAA
c-Abli	GACCAACUUGUUCAGCGCC
RAD51i	GCAGUGAUGUCCUGGAUAA
RAD52I	GGUCCAUGCCUUUAAUGUUTT
LIG4 i	AAGCCAGACAAAAGAGGUGAA
siNS	UUCUCCGAACGUGUCACGUTT
